# Supplementary material for: Tumor-targeted silencing of the peptide transporter TAP induces potent antitumor immunity
Source: Nat Commun. 2019 Aug 21;10:3773. doi: 10.1038/s41467-019-11728-2 (PMC6704146; doi:10.1038/s41467-019-11728-2)
Supplement: Supplementary file 1 — Supplementary Information [file 41467_2019_11728_MOESM1_ESM.pdf]

SUPPLEMENTARY FIGURES & TABLES

Tumor-targeted silencing of the peptide transporter TAP induces potent antitumor immunity

Greta Garrido[1], Brett Schrand[1], Ailem Rabasa[1], Agata Levay[1], Francesca D'Eramo[1], Alexey Berezhnoy[1], Shrey Modi[2], Tal Gefen[1], Koen Marijt[3], Elien Doorduijn[3], Vikas Dudeja[2], Thorbald van Hall[3], Eli Gilboa[1]*

Department of Microbiology and Immunology1, Department of Surgery2, University of Miami, Miller School of Medicine, Miami, FL, Department of Medical Oncology3, Leiden University Medical Center, Leiden, Netherlands.

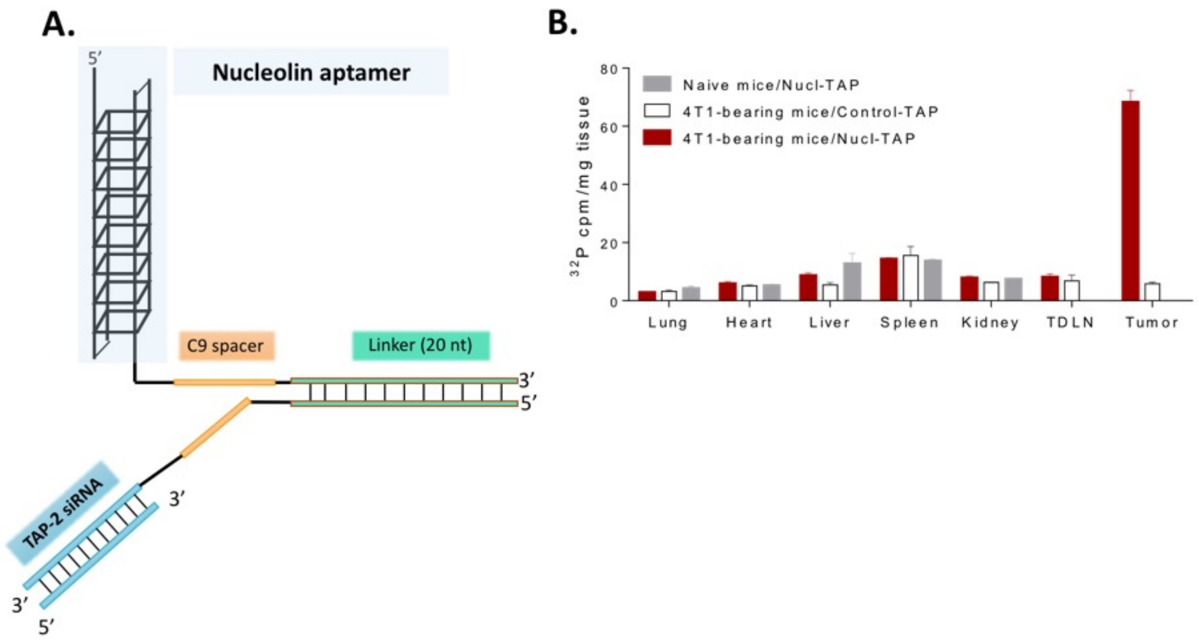

Supplementary Fig.1. Accumulation of systemically administered nucleolin aptamer-shRNA conjugates in tumors. **A.** Structure of a 26 nt G-rich G-quartet forming nucleolin aptamer[17] conjugated to a TAP siRNA. **B.** ^{32}P-labeled nucleolin or a non-binding aptamer conjugated to TAP-shRNA were administered via intraperitoneal to subcutaneously implanted 4T1 tumor bearing mice or the nucleolin aptamer was administered to tumor-free mice, and 24 h later tumor and indicated organs were excised and radioactivity was measured using a scintillation counter. Data represent means ± SEM (4 mice/group).

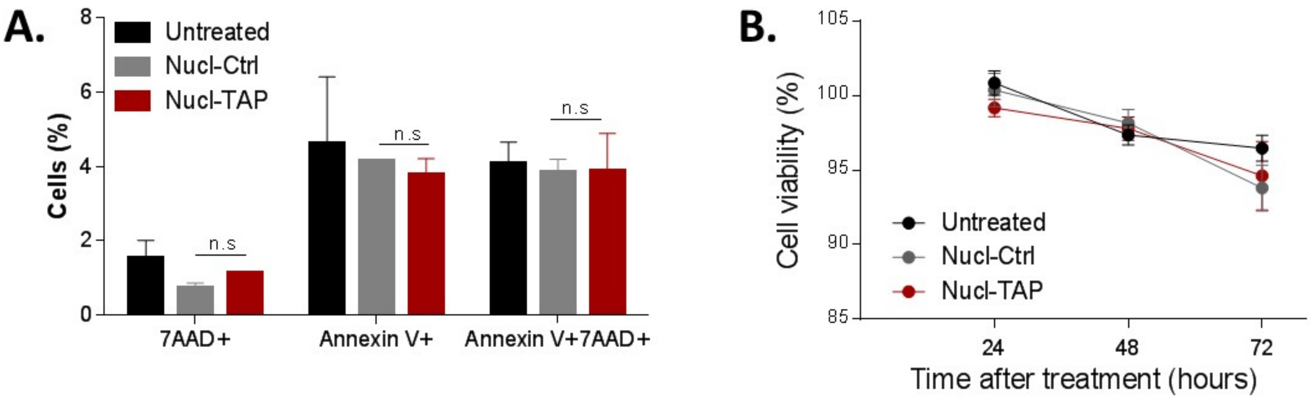

Supplementary Fig. 2. Toxicity of Nucl-TAP or Nucl-Ctrl siRNAs in cultured tumor cells. 4T1 tumor cells were cultured in the presence of 0.5 µM aptamer conjugates (n=2). **A.** Apoptosis. After 24 h was determined by staining with 7AAD and Annexin-V, and analyzed by flow cytometry. **B.** Cell viability was determined by MTT assay 1, 2 and 3 days after aptamer-siRNA conjugate treatment. Data represent means ± SEM of triplicate wells.

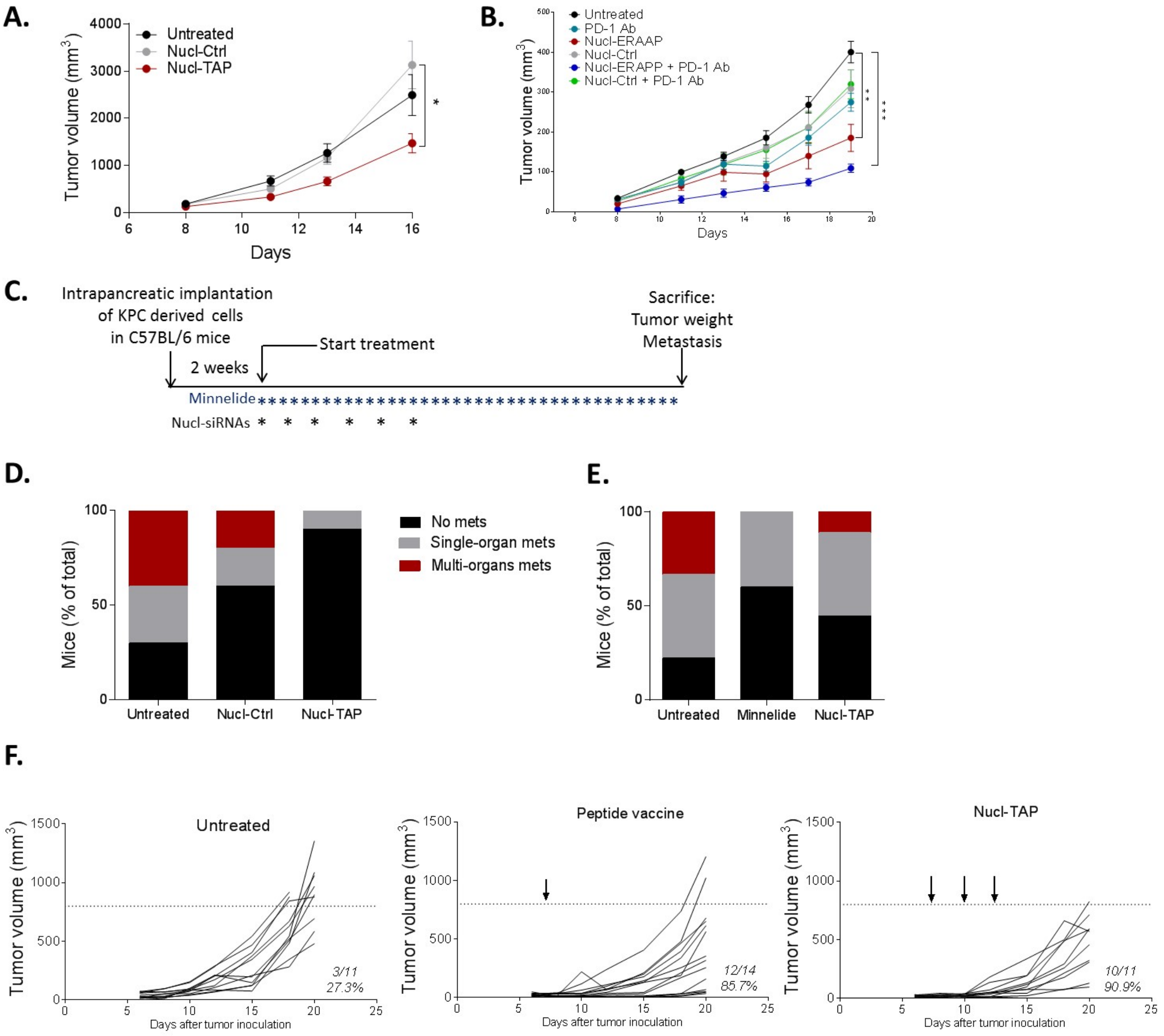

Supplementary Fig. 3. Nucleolin aptamer-targeted TAP siRNA mediated inhibition of tumor growth in mice. **A.** RMA T lymphoma model. Subcutaneously implanted palpable day 7 RMA tumor bearing C57BL/6 mice were administered i.p. with Nucl-TAP or Nucl-Ctrl, repeated two additional times 3 days apart, and tumor growth was monitored. (8-9 mice/group) (n=4). **B.** Nucleolin aptamer-targeted ERAAP siRNA mediated inhibition of tumor growth in mice – synergy with anti-PD-1 antibody treatment. Subcutaneously implanted palpable day 8 4T1 tumor bearing Balb/c mice were administered intraperitoneally with Nucl-ERAAP or Nucl-Ctrl followed by PD-1 antibody a day later, repeated two additional times 3 days apart, and tumor growth was monitored. (7-13 mice/group) (n=2). Data are represented as mean ± SEM. **C-E**. Nucl-TAP mediated inhibition of metastasis in the KPC-derived pancreatic cancer model. Each column represents the metastatic burden per group. **C**. Experimental protocol. See Methods & details in Fig. 3D & E. **F**. Nucl-TAP mediated inhibition of tumor growth in the MC38 tumor model. Experiments as shown in Fig. 3G, except that data are presented for individual mice as shown in reference[21]. Arrows indicate treatment frequency for each group.

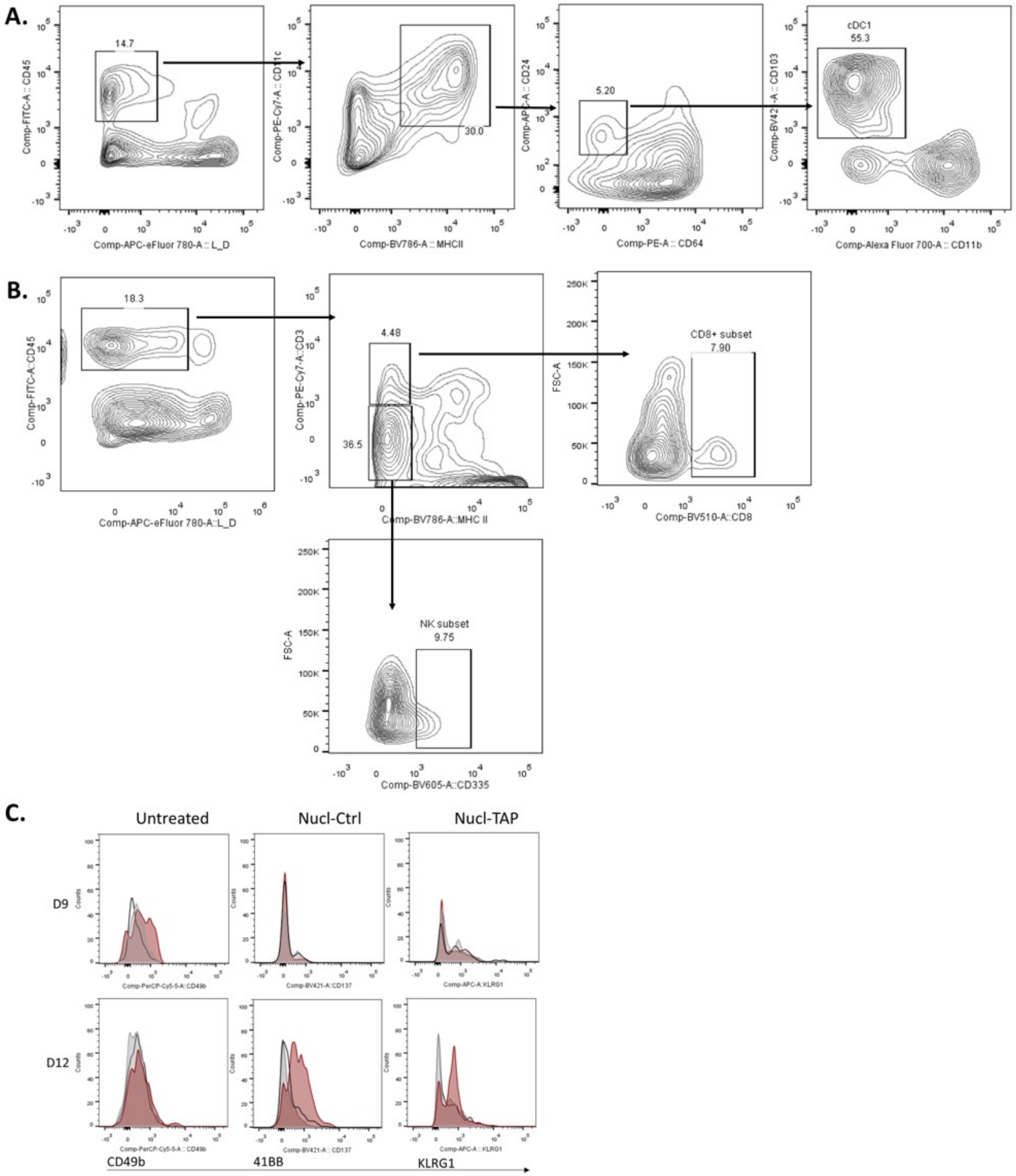

Supplementary Fig. 4. NK, CD8+ T cells and cDC1 accumulate in the TME of Nucl-TAP treated tumor bearing mice. 4T1-bearing mice were injected i.p. with Nucl-siRNA conjugates as described in Fig.3A and infiltrating immune cells were analyzed 2 days after the first and second dose by flow cytometry. Gating strategy to identify intratumoral NK and CD8+T cells **(A)** or cDC1 **(B)**. Numbers represent % cells within depicted gate. **C.** Analysis of intratumoral NK cells for maturation and activation markers, representative tumor.

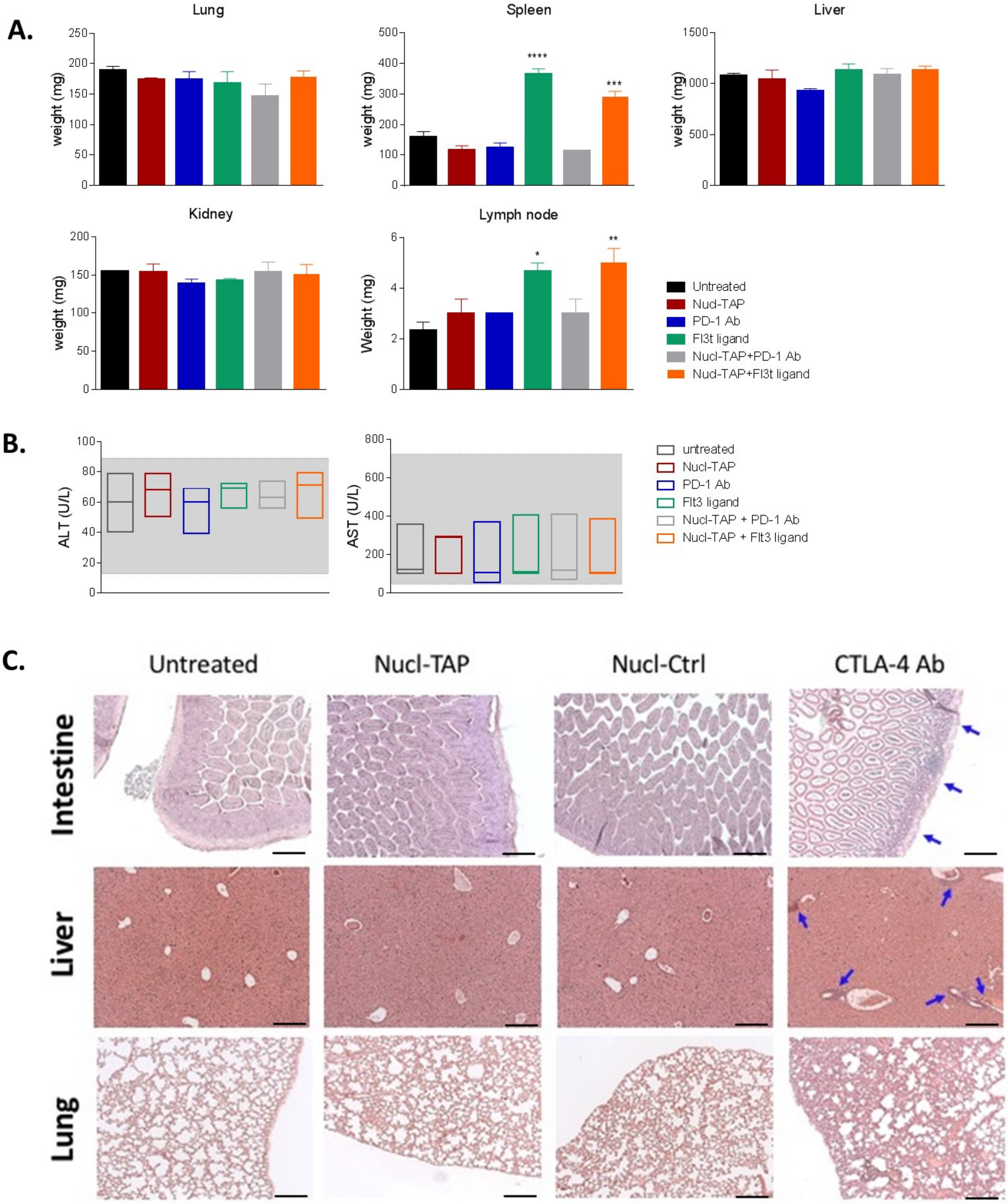
A.
Lung
Spleen
Liver
Kidney
Lymph node
weight (mg)
Weight (mg)
****
***
*
**
Untreated
Nud-TAP
PD-1 Ab
Fl3t ligand
Nucl-TAP+PD-1 Ab
Nud-TAP+Fl3t ligand
B.
ALT (U/L)
AST (U/L)
untreated
Nucl-TAP
PD-1 Ab
Flt3 ligand
Nucl-TAP + PD-1 Ab
Nucl-TAP + Flt3 ligand
C.
Untreated
Nucl-TAP
Nucl-Ctrl
CTLA-4 Ab
Intestine
Liver
Lung

Supplementary Fig. 5. Toxicity in mice treated with Nucl-TAP. Balb/c mice (3 mice/group) were administered with Nucl-TAP, Nucl-Ctrl, PD-1 Ab, or Flt3 ligand as indicated using doses and regimen used in the immunotherapy studies. Two days post last administration mice were sacrificed and analyzed (see also Fig. 5F). **A.** Organ weight. Data are represented as means ± SEM. **B.** Liver enzymes AST and ALT in the serum. Shaded area represents normal levels of ALT or AST in Balb/c mice (from The Jackson Laboratories (MPD, http://phenome.jax.org/). Data represent box plot analysis. No statistical differences were found between groups using the Kruskal-Wallis test with Dunn posttest. **C.** Inflammatory responses in tissue sections stained with hematoxylin and eosin and visualized by light microscopy at 10X magnification (Scale bar: 100 µm). One group of mice was also treated with 200 µg of CTLA-4 antibody that elicits a comparable antitumor effect[27,28]. Arrows indicate inflammatory foci in mice.

Variable	Treatment groups (4 mice/group)		
	Untreated	Nucl-Ctrl	Nucl-TAP
White Blood Cell Count (WBC) (10^3 cells/µl)	4.22 ± 0.26	4.87 ± 0.50	3.65 ± 0.17
Red Blood Cell Count (RBC) (10^3 cells/µl)	9.36 ± 0.30	9.32 ± 0.20	9.95 ± 0.61
Hemoglobin (g/DL)	14.12 ±0.33	13.72 ± 0.28	14.4 ± 0.52
Hematocrit (%)	56.00 ± 3.16	54.00 ± 1.41	55.5 ± 1.91
Mean Cell Volume (MCV) (fL)	59.75 ± 2.06	57.75 ± 1.70	56.5 ± 1.00
Mean Cell Hemoglobin (MCH) (pg)	15.00 ± 0.00	14.50 ± 0.57	14.50 ± 0.57
Mean Cell Hemoglobin Concentration (MCHC) (%)	25.50 ± 1.00	25.25 ± 0.95	26.00 ± 1.41
Segmented Neutrophils (10^3/µl)	19.00 ± 4.54	15.50 ± 1.29	17.50 ± 0.57
Lymphocytes (10^3/µl)	72.75 ± 2.87	75.75 ± 2.21	72.00 ± 1.70
Monocytes (10^3/µl)	8.25 ± 2.21	8.5 ± 1.29	10.00 ± 1.50
RBC Morphology	Mild Polychromasia	Mild Polychromasia	Mild Polychromasia
Platelet Morphology	Normal	Normal	Normal
WBC Morphology	Normal	Normal	Normal

Supplementary Table 1. Complete blood count analysis in Bab/c mice treated with Nucl-siRNA conjugates. Untreated mice were used as internal control (normal values for Balb/c mice from The Jackson Laboratory: http://phenome.jax.org/). Values are represented as mean ± SD. No statistical differences were found comparing Nucl-siRNA conjugates treated mice to controls using the Kruskal–Wallis test with Dunn posttest.
